# Modulation of pain sensitivity by tDCS using different anodal connector locations: a single-blinded, randomized, sham-controlled study

**DOI:** 10.3389/fpain.2025.1533962

**Published:** 2025-06-16

**Authors:** Shang-Yueh Tsai, Yi-Ru Lin, David M. Niddam

**Affiliations:** ^1^ Graduate Institute of Applied Physics, National Chengchi University, Taipei, Taiwan; ^2^ Research Center for Mind, Brain and Learning, National Chengchi University, Taipei, Taiwan; ^3^ Department of Electronic and Computer Engineering, National Taiwan University of Science and Technology, Taipei, Taiwan; ^4^ Brain Research Center, National Yang Ming Chiao Tung University, Taipei, Taiwan; ^5^ Institute of Brain Science, National Yang Ming Chiao Tung University, Taipei, Taiwan; ^6^ Institute of Neuroscience, National Yang Ming Chiao Tung University, Taipei, Taiwan

**Keywords:** GABA, pain threshold, sensorimotor cortex, transcranial direct current stimulation, multi-session, long-lasting effect

## Abstract

**Background:**

The efficacy of transcranial direct current stimulation (tDCS) depends on various stimulation parameters. With rectangular electrodes, the location of the wire connector may affect the electrical field relative to the underlying target area. Here, we examined longitudinal changes in pain sensitivity and GABA levels in response to tDCS using standard rectangular (5 × 7 cm) electrodes and two different anodal connector locations.

**Methods:**

In this single-blinded, randomized, sham-controlled study, 53 healthy volunteers were assigned to one of 4 groups, receiving either real tDCS or sham tDCS, with the anodal connector oriented either superior-medially or ventral-laterally. tDCS was delivered on 5 consecutive days with the anode and cathode placed over the left primary sensorimotor cortex (SM1) and the right dorsolateral prefrontal cortex, respectively. Pain detection thresholds (PT) and moderate pain thresholds (MPT) of the right index finger and GABA levels from the bilateral SM1 were obtained prior to tDCS, after 5 tDCS sessions, and after 6 weeks.

**Results:**

Superior-medial oriented tDCS significantly increased both pain thresholds at day 5 and at 6 weeks, whereas ventral-lateral oriented tDCS or sham tDCS did not. At day 5, MPT was significantly increased when comparing superior-medial oriented tDCS with sham tDCS. At week 6, both thresholds were significantly increased when comparing superior-medial oriented tDCS with ventral-lateral oriented tDCS and MPT was also increased when comparing superior-medial oriented tDCS with sham tDCS. GABA levels did not differ between time-points or between groups and no association was found between baseline GABA levels in the stimulated hemisphere and change in pain thresholds.

**Conclusions:**

tDCS-induced long-lasting changes in pain sensitivity may depend on the location of the wire connector when using a rectangular anode. A greater pain modulatory effect may be induced when the connector is aligned superior-medially along the central sulcus.

## 1 Introduction

Transcranial direct current stimulation (tDCS) holds potential as a safe and non-invasive technique for modulating pain perception (1–3). By targeting specific brain regions involved in pain processing, such as the primary sensorimotor cortex (SM1) or the dorsolateral prefrontal cortex (DLPFC), tDCS can reduce the perceived intensity of acute pain in healthy individuals (2, 4, 5). tDCS modulates neuronal excitability by delivering low-intensity electrical currents to the underlying cortical areas. Different electrode montages of tDCS have been associated with polarity specific effects on cortical excitability, leading to excitation (anodal tDCS) or inhibition (cathodal tDCS) (6). These changes in excitability can persist beyond the duration of a single stimulation session (6, 7). Moreover, multiple tDCS sessions on consecutive days can prolong the effect on clinical pain for weeks or even months (8–10).

The efficacy of tDCS is influenced by various stimulation parameters (11). However, little attention has been paid to the impact of electrode orientation relative to the underlying target area. Different orientations may induce varying levels of excitability changes (12). Moreover, the highest current density is likely to occur near the wire connector's entry point rather than at the geometric center of the electrode (13). This issue is particularly relevant when using rectangular electrodes, as the center of the electrode may not align with the region of peak current density. Since few studies report the precise location of the wire connector when employing standard rectangular electrodes, this factor could contribute to the variability in efficacy observed across studies (14).

Single-session anodal tDCS targeting the primary motor cortex (M1) has been shown to immediately reduce GABA levels in the underlying cortex (7, 15–18). Since GABA levels in bilateral SM1 have also been associated with pain sensitivity (19), it is possible that baseline GABA levels, or the modulation of GABA, may underlie the effects of tDCS on pain sensitivity. In this study, we investigated long-lasting changes in pain sensitivity and GABA levels in response to multi-session anodal tDCS targeting the left SM1. The anode was oriented with the wire connector pointing either superior-medially (tDCS-Top) or ventral-laterally (tDCS-Bottom). We hypothesized that (1) pain sensitivity would decrease following anodal tDCS using both electrode orientations relative to sham stimulation, and (2) the response to tDCS would differ between the two electrode orientations due to the targeting of slightly different cortical areas. As pain sensitivity may be both trait and state dependent, we further hypothesized that (3) better long-lasting outcomes would be associated with lower baseline GABA levels (reflecting reduced inhibition) in the stimulated hemisphere; and (4) GABA levels would change in the stimulated cortex (left SM1) but not in the non-stimulated cortex (right SM1) following anodal tDCS. Finally, we simulated the electric field and current density distributions for the two tDCS configurations in a standard head model.

## 2 Materials and methods

### 2.1 Study design

**Figure 1:**
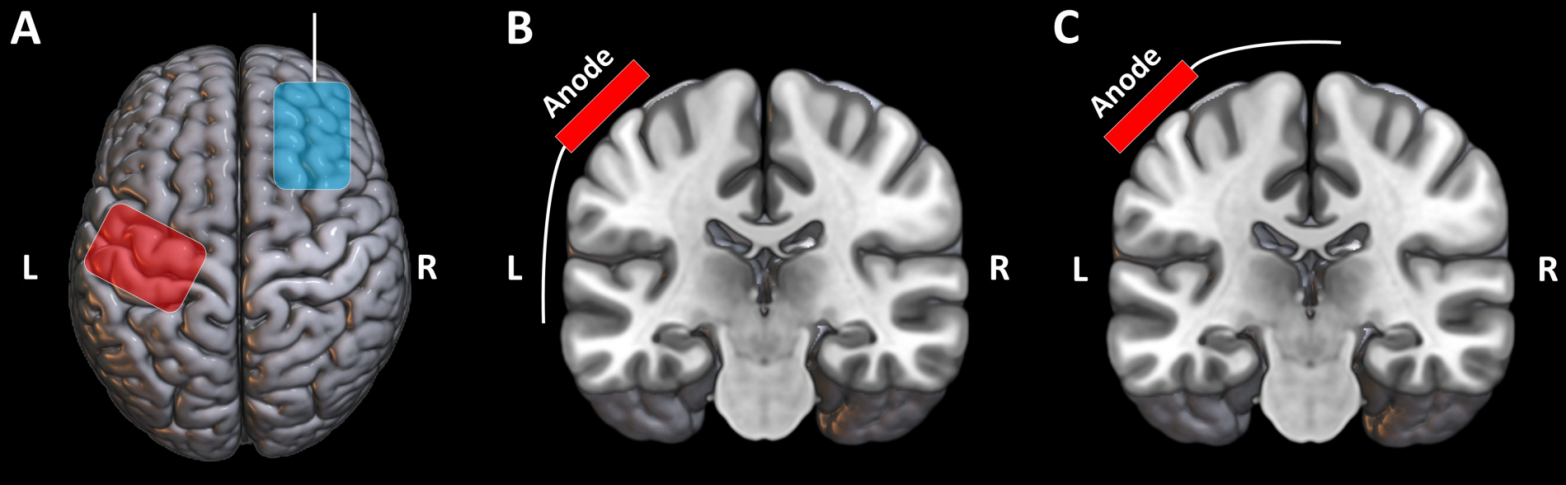
Schematic diagram of the orientation of the electrodes. **(A)** Top view showing the connector location of the cathode. **(B)** Coronal view showing the connector location of the anode for tDCS-Bottom. **(C)** Coronal view showing the connector location of the anode for tDCS-Top. The diagram is for illustrative purpose only.

In this single-blinded, randomized, sham-controlled, longitudinal study, healthy participants underwent 5 tDCS sessions, one per day, on consecutive days. tDCS was applied with rectangular electrodes in all participants but with different orientations of the wire connector. The study was conducted as a parallel trial, with computerized stratified randomization (1:1:1:1) used to allocate participants to one of four groups: (1) anodal tDCS with the wire connector aligned superior-medially (tDCS-Top), (2) anodal tDCS with the wire connector aligned ventral-laterally (tDCS-Bottom), (3) sham tDCS with the wire connector aligned superior-medially, and (4) sham tDCS with the wire connector aligned ventral-laterally (Figure 1). Stratification was based on 6 strata, defined by sex (male/ female) and age group (18–30, 31–40, 41–50). DMN was responsible for generating the allocation sequence and assigning participants to groups. Participants were blinded to their group allocation, including the orientation of wire connectors and whether they received real or sham tDCS. They were informed that the study would investigate the long-term effect of tDCS on acute pain. All participants were naïve to tDCS and were fully debriefed after the experiment. Psychological assessment with questionnaires was performed just prior to the first tDCS session (baseline, denoted as D1). Pain thresholds were assessed at D1, after the fifth tDCS session (denoted as D5) and 6 weeks after the first tDCS session (denoted as W6). Magnetic resonance imaging was also performed at D1 before tDCS, at D5 after tDCS and at W6, each occurring after threshold assessment. The decision to conduct follow-up at W6 was informed by findings from pilot experiments, which suggested that the maximal modulatory effect on pain thresholds with the applied tDCS protocol was observed around the sixth week. Psychological and psychophysical assessments, as well as tDCS, were carried out in a quiet, climate-controlled, soundproof room located at National Yang Ming Chiao Tung University. During this part of the experiment, participants sat in a comfortable, padded chair with armrests, while the stimulators were positioned behind them, out of their line of sight. Since we considered our study exploratory in terms of methodology, e.g., we did not specify which electrode orientation would produce the largest effect size, and the combination of tDCS parameters applied has not previously been reported, we did not preregister it.

### 2.2 Participants

The sample size was based on the number of participants that could be recruited and assessed within the study timeline (August 2013–July 2016). All participants were recruited by a research assistant assigned to the project. In total, 55 healthy volunteers participated in the study. All participants were required to be over 18 years old and right-hand dominant as assessed with the Edinburgh Handedness Inventory for daily activities (laterality score ≥ 0.75) (20). Exclusion criteria were: (1) implanted electronic devices; (2) metal implants in the head; (3) skin lesions or damage at the electrode sites (head and finger); (4) a history of neurological (e.g., stroke, epilepsy) or psychiatric disorders or head trauma; and (5) pregnancy. The study received approval from the Institutional Review Board of Taipei Veterans General Hospital (VGH 2013-06-009A), and all participants provided written, informed consent in accordance with the Declaration of Helsinki (6th revision).

### 2.3 Psychological and psychophysical assessments

All questionnaires and pain tests were administered by the same operator. The psychological state of the participants was assessed using the Pain Catastrophizing Scale (PCS) (21), the Spielberger State-Trait Anxiety Inventory (STAI) (22) and the Center for Epidemiological Studies-Depression Scale (CES-D) (23).

The Pain Catastrophizing Scale was used to evaluate the extent to which a person experiences catastrophic thinking in relation to pain**.** It is divided into three subscales: rumination, magnification, and helplessness. The scale consists of 13 items, each rated on a scale from 0 (“Not at all”) to 4 (“all the time”). The total score ranges from 0 to 52, with a higher score indicating a greater tendency toward catastrophic thinking in response to pain. The scale was originally validated in healthy participants exposed to experimental pain and in a clinical population undergoing medical procedures (21). The Chinese version of the scale (HK-PCS) has shown high internal consistency and a strong test-retest reliability (24). The STAI questionnaire was used to assess both state anxiety (temporary) and trait anxiety (predispositional). Each subscale consists of 20 items, which are rated on a scale from 1 (“Not at all”) to 4 (“Very much so”), with total scores ranging from 20 to 80. A higher score indicates a greater level of anxiety, and a score below 40 is considered to indicate low anxiety. The Chinese version of the questionnaire has high internal consistency and excellent test-retest reliability (25, 26). The CES-D scale was used to measure participants' depressive symptoms over the past week based on symptom frequency. It consists of 20 items, each rated on a scale from 0 (“rarely” or under 1 day) to 3 (“Most or all the time” or 5–7 days), with total scores ranging from 0 to 60. A higher score indicates more severe depressive symptoms, while scores under 16 indicate non-clinical levels of symptoms. The scale has been widely applied in both general and psychiatric populations, demonstrating high internal consistency and adequate test-retest reliability (23). The Chinese version of the scale has also shown good internal consistency and excellent test-retest reliability (27, 28).

Prior to the pain tests, the operator carefully explained the testing procedures to the participants to ensure compliance. During the pain tests, participants rested their lower right arm on the armrest while electrical stimulation (0.2 ms square wave pulse; Digitimer DS-7A, Hertfordshire, England) was applied to the palmar surface of the distal phalanx of the right index finger. A bipolar probe (25 mm inter-electrode distance, Grass Technologies Corp., USA), with the anode placed distally, was used for stimulation. Stimulus intensities corresponding to the pain detection threshold (PT) and moderate pain threshold (MPT) were then registered using the method of ascending limits in four series (with the first series discarded). The stimulus delivery was computer-controlled and stimuli were separated by an interval of 15 s. Participants were instructed to verbally indicate as soon as they perceived each of the two levels. The perception of a sharp pricking sensation is thought to correspond to Aδ-fiber activation (29). No rating scale was needed for PT. However, for the moderate pain intensity, an 11-point numerical pain intensity rating scale was used with the anchor points “0” and “10” corresponding to “no pain” and “worst possible pain”, respectively. Moderate pain was defined as “5” on the scale [see, e.g., Table 3 in (30)]. Suprathreshold pain intensity was applied because pain detection thresholds may be less relevant in clinical settings.

### 2.4 Imaging acquisition

Magnetic resonance imaging and spectroscopy data were acquired on a 3T system (Trio, Siemens Medical Solutions, Erlangen, Germany) with a 32-channel head coil array. Participants were instructed to lie in the scanner with their eyes closed and to refrain from moving their heads during the experiment. Initially, a high-resolution 3D MPRAGE (Magnetization Prepared Rapid Acquisition Gradient Echo) anatomical scan (repetition time [TR]/echo time [TE]/flip angle [FA]: 2,530 ms/3.03 ms/7 degrees; field of view (FOV): 224 × 256 × 192; voxel size: 1 × 1 × 1 mm^3^) was acquired.

The volumes of interests (VOIs) for magnetic resonance spectroscopy (MRS) were first delineated on the anatomical image. VOIs (25 × 25 × 25 mm^3^) were placed over the left and right hand representations of SM1 according to known landmarks, i.e., the precentral knob in the axial plane and the hook-shaped sulcus in the sagittal plane (19). The VOIs were rotated in the sagittal, axial and coronal planes to align the edges parallel with the surface of the cortex. This ensured maximum coverage of the hand area. An MRS pre-scan was then carried out using a point resolved spectroscopy (PRESS) sequence (TR/TE = 2,000/68 ms, sample points = 2,048, bandwidth = 2,000 Hz, 8 averages). These spectra were analyzed and displayed online to assess linewidth, water suppression, and noise level. To be deemed of acceptable quality, the following criteria had to be met: (1) absence of lipid contamination (due to the proximity of the VOIs to the cortical surface); (2) clear separation between creatine and choline peaks; (3) N-acetylaspartate linewidth estimated on the console of less than 10 Hz; and (4) absence of high-frequency noise. Once spectra in the pre-scan were considered to be of acceptable quality, GABA measurements were then performed using the same adjustment parameters for shimming, resonance frequency, and water suppression. For GABA measurements, a MEGA-PRESS sequence was employed (31). A total of 300 spectra were acquired in 75 dynamic scans with 4-step phase cycling using the following parameters: TR/TE = 2,000/68 ms, sample points = 2,048, bandwidth = 2,000 Hz. GABA-editing was achieved with a 15 ms Gaussian pulse applied at 1.9 ppm for edit-on spectra and at 7.5 ppm for edit-off spectra. The editing pulse (off/on) was applied in an alternating sequence with each dynamic scan.

### 2.5 tDCS protocol

**Figure 2:**
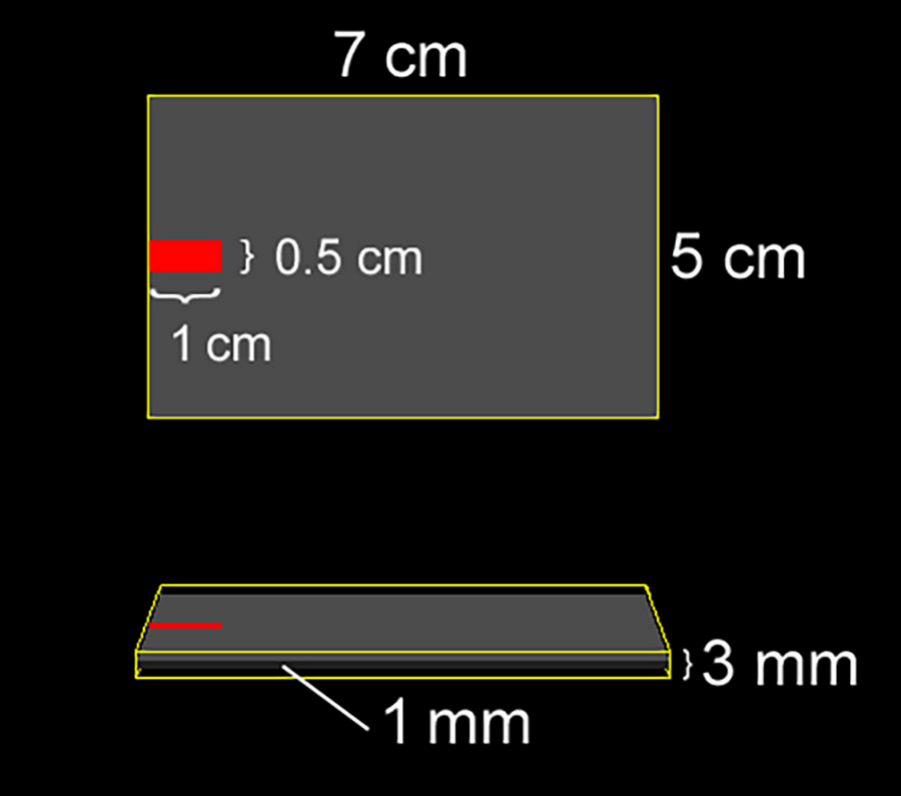
Geometry and dimensions of the electrode used in the simulation. (Top) View of the electrode from above; (Bottom) Side view. The rubber pad (grey) is embedded within a saline-soaked sponge (yellow), and the connector is positioned at the distal end of the electrode, centered along its width.

The DC-Stimulator Plus (neuroConn GmbH, Ilmenau, Germany) was used for non-invasive cortical stimulation. Stimulation electrodes consisted of a pair of rectangular rubber electrodes, each embedded in color-coded, saline-soaked (0.9% NaCl) sponges with an area of 35 cm^2^ (5 cm × 7 cm). The center of the anode was placed over the left M1 (C3 location according to the 10–20 system), with the long side of the electrode oriented at a 45-degree angle to the mid-sagittal plane. The anode was rotated 180 degrees between the “Top” and “Bottom” configurations. The connection point between the electrode and the connector cable was at the far end of the rectangular electrode, at the midpoint of the width, as indicated in Figures 1A, 2. The center of the cathode was placed over the right DLPFC (F4 location), with the wire pointing anteriorly and the long axis of the electrode aligned parallel to the midline (Figures 1, 2). The connections consisted of a socket on the electrode and a pin (1.5-mm diameter) on the connection cable (touch-proof DIN 42802-2).

The decision to apply cathodal stimulation over the right DLPFC was informed by both the valence lateralization hypothesis (32, 33) and existing literature on non-invasive brain stimulation in major depression and chronic pain. According to the valence lateralization hypothesis, the left and right prefrontal cortices are differentially involved in emotional processing—positive (or approach-related) emotions are associated with the left, and negative (or withdrawal-related) emotions with the right prefrontal cortex. In line with this, inhibitory repetitive transcranial magnetic stimulation over the right DLPFC has been used to reduce hyperactivity in this region and alleviate negative affect in patients with major depression (34). A similar approach has been applied in tDCS studies, particularly using bilateral DLPFC stimulation (anodal left/cathodal right) (35). Importantly, this principle has also been extended to chronic pain, where inhibitory rTMS of the right DLPFC and bifrontal tDCS (anodal left/cathodal right) have demonstrated therapeutic potential (36–38). Taken together, since pain is closely linked to negative affect, targeting the right DLPFC may reduce pain unpleasantness by dampening activity in this region.

An intermittent tDCS protocol was employed (39, 40), involving daily M1-tDCS sessions (intensity = 2 mA) conducted over 5 consecutive days. Each session consisted of two 10 min tDCS blocks, separated by a 10 min rest period. Sham stimulation was delivered using the same stimulus intensity and rectangular electrodes, but with a stimulus duration of 30 s applied only once at the beginning of the session. As mentioned above, participants were allocated to one of four groups in which real tDCS or sham tDCS was performed with the anodal electrode wire oriented either superior-medially or ventral-laterally (Figure 1). For all types of stimulation and blocks, the current was ramped up and down over 30 s

### 2.6 Spectral analysis

Spectral processing of the MEGA-PRESS data was conducted using MATLAB R2023a (MathWorks, Natick, USA). The initial dynamic scan, comprising four spectra, was excluded. Subsequently, the remaining 74 dynamic scans (296 spectra) were averaged within each dynamic scan after correcting for frequency drift based on the peak of total creatine (tCr; including creatine and phosphocreatine) at 3.0 ppm. This process resulted in 37 edit-on spectra and 37 edit-off spectra. The 74 spectra were then analyzed and quantified using the Gannet 3.1 toolbox (41), an open-source MATLAB-based software package. Five modules—GannetLoad, GannetFit, GannetCoRegister, GannetSegment, and GannetQuantify—were employed for spectral processing. In short, spectral phase registration and frequency correction were applied with a line broadening of 3 Hz and zero padding by a factor of 16. The GABA signal at 3.0 ppm and Glx (combined glutamate and glutamine) at 3.75 ppm in the edited spectrum were quantified by fitting a triple Gaussian model with a linear baseline and sinusoidal terms across a range from 2.79 ppm to 4.10 ppm. The unsuppressed water signal was modeled using a Lorentzian-Gaussian function. To account for relaxation and tissue type, correction factors were applied using subject-specific voxel masks generated for grey matter, white matter, and cerebrospinal fluid fractions. The estimated GABA levels in institutional units (IU) relative to the water signal were derived following tissue-specific water visibility and relaxation corrections (T1 and T2) using the Gasparovic method (42). In this study, the quantified GABA concentrations were denoted as GABA+, as they also encompass contributions from homocarnosine and macromolecules. Spectra with a normalized residual fitting error of GABA+ above 8% and a linewidth of the unsuppressed water signal exceeding 13 Hz (approximately 0.1 ppm) were excluded from the final analysis to ensure data quality. Additionally, data quality was evaluated through visual inspection to identify and discard spectra displaying strongly aberrant features within the fit range, adhering to data quality recommendations (43).

### 2.7 Simulation of electric fields and current densities

The SimNIBS 4.5 software package (11, 44) was used to simulate electric fields and current density magnitudes. The software includes a standard head model based on the finite element method, constructed from T1- and T2 weighted magnetic resonance images of a single individual. The head model was automatically generated, and default isotropic conductivities were assigned to different tissue classes as follows: white matter (0.126 S/m), grey matter (0.275 S/m), cerebrospinal fluid (1.654 S/m), scalp (0.465 S/m), eyeballs (0.5 S/m), compact bone (0.008 S/m), spongy bone (0.025 S/m), blood vessels (0.600 S/m), and muscle (0.160 S/m). Electrodes were modeled as rectangular rubber pads (5 cm × 7 cm, thickness: 1 mm, conductivity: 29.4 S/m) embedded in saline-soaked sponges (5 cm × 7 cm, thickness: 3 mm, conductivity: 1.0 S/m) (Figure 2). The connector (1 cm long, 0.5 cm wide) was positioned at the far end of the electrode, centered along its width (Figure 2). The anode was placed over the C3 location, rotated 45 degrees relative to the mid-sagittal plane, and assigned a current of +2 mA. “Top” and “Bottom” montages were simulated by varying the connector's position at either end of the electrode. The cathode was placed over the F4 location, aligned with the midline of the scalp, and assigned a current of −2 mA, with the connector positioned anteriorly.

### 2.8 Study outcomes

The study analyzed longitudinal changes in pain thresholds and GABA levels. The primary outcome measures were the pain detection threshold (PT) and suprathreshold pain corresponding to moderate pain intensity (MPT). These measures were assessed within groups using untransformed values, and between groups using changes relative to the baseline.

Our analyses proceeded according to the two main hypotheses. First, to test whether pain sensitivity would decrease following anodal tDCS, we conducted both within-group and between-group analyses, regardless of wire connector orientation. Second, to test whether the response to tDCS differed between electrode orientations, participants were further divided according to the wire connector orientation.

Secondary outcome measures included GABA+ concentrations in the stimulated (left) and unstimulated (right) SM1. To assess whether GABA levels would change in the stimulated cortex but not in the non-stimulated cortex following anodal tDCS, the analysis proceeded as for thresholds. Additionally, we investigated whether greater long-term improvement (i.e., larger changes in thresholds) was associated with lower baseline GABA levels in the stimulated cortex (left SM1).

### 2.9 Statistical analyses

Statistical analyses were conducted using SPSS (IBM SPSS, version 21.0). The data were initially tested for normality using the Shapiro–Wilk test, which was applied separately to each group (tDCS and Sham), sub-group (tDCS-Top, tDCS-Bottom, Sham-Top, and Sham-Bottom), outcome, and time point. As all data in the tDCS group and sub-groups, and some data in the Sham-Bottom group, were found to be non-normally distributed, non-parametric tests were selected to ensure consistency across groups. Each group's data were further examined for outliers. Given the substantial individual variability in pain sensitivity, outliers were defined as values exceeding three times the interquartile range above or below the upper and lower quartiles within each sub-group. When an outlier was identified for a specific threshold in a participant, all time points for that threshold in that participant were removed, while data for the other threshold were retained. Because the primary outcomes in the between-group analysis were based on relative values, an outlier in the within-group analysis did not necessarily imply an outlier in the between-group analyses, and vice versa. All within- and between-group comparisons for the primary (PT and MPT) and secondary (GABA+ levels) outcomes were conducted with outliers excluded.

In analyses where wire connector orientation was not considered, within-group comparisons across time points (D1, D5, W6) were made separately for PT and MPT using Friedman tests (PT tDCS, MPT tDCS, PT Sham, and MPT Sham). Tests that met the Bonferroni-adjusted significance threshold (4 tests, *p* = 0.0125) were followed by *post-hoc* Dunn's tests, with further Bonferroni adjustment for three pairwise comparisons (D1 vs. D5, D1 vs. W6, D5 vs. W6; *p* = 0.0167). Baseline between-group comparisons for PT and MPT were performed separately using Mann–Whitney *U*-tests (2 tests, adjusted *p* = 0.0250). Changes in PT and MPT relative to the baseline (D5 vs. D1 and W6 vs. D1) were also compared between groups using Mann–Whitney *U*-tests (4 tests, adjusted *p* = 0.0125).

To examine the effect of wire connector orientation, the tDCS group was further divided into tDCS-Top and tDCS-Bottom sub-groups. To enhance statistical power, and given prior within-group analyses found no differences across time points in the Sham group, the Sham group was not further subdivide, which is justified as sham stimulation is not expected to induce threshold changes. Within-group comparisons across time points were again made separately for each threshold and group using Friedman tests (PT tDCS-Top, MPT tDCS-Top, PT tDCS-Bottom, MPT tDCS-Bottom). Tests passing the Bonferroni-adjusted threshold (4 tests, *p* = 0.0125) were followed by *post-hoc* Dunn's tests with further adjustment (D1 vs. D5, D1 vs. W6, D5 vs. W6; *p* = 0.0167). Because the Sham group remained unchanged, its within-group analysis was not repeated. Between-group comparisons at baseline were assessed using the Kruskal–Wallis test for PT and MPT, separately (2 tests, adjusted *p* = 0.0250). Between-group comparisons of threshold changes relative to the baseline were also made using the Kruskal–Wallis test (PT: D5 vs. D1 and W6 vs. D1; MPT: D5 vs. D1 and W6 vs. D1; 4 tests, adjusted *p* = 0.0125). Tests passing a Bonferroni-adjusted threshold were again followed by *post-hoc* Dunn's tests with further adjustment (tDCS-Top vs. tDCS-Bottom, tDCS-Top vs. Sham, tDCS-Bottom vs. Sham; *p* = 0.0167).

For the analyses of GABA+ concentrations, within-group comparisons across time points were made separately for the left and right hemispheres in each tDCS group using Friedman tests, applying Bonferroni-adjusted thresholds (6 tests, *p* = 0.0083). Between-group comparisons of baseline GABA+ levels were conducted using the Kruskal–Wallis test for each hemisphere. Between-group comparisons of GABA+ changes at D5 and W6 were also conducted separately by hemisphere using Kruskal–Wallis tests (4 tests, *p* = 0.0125). *post-hoc* tests followed the same procedure as for threshold outcomes. Finally, to assess whether baseline GABA+ levels in the stimulated hemisphere (left SM1) were predictive of outcome, Spearman's rank correlation analyses were performed between left GABA+ concentrations and pain threshold changes at time-points with significant between-group differences. Given the *a priori* hypotheses, one-tailed *p*-values were used with adjustment for the multiple tests (6 tests: PT tDCS-Top, MPT tDCS-Top, PT tDCS-Bottom, MPT tDCS-Bottom, PT Sham, MPT Sham; adjusted *p* = 0.0083).

## 3 Results

### 3.1 Characteristics of the participants

**Figure 3:**
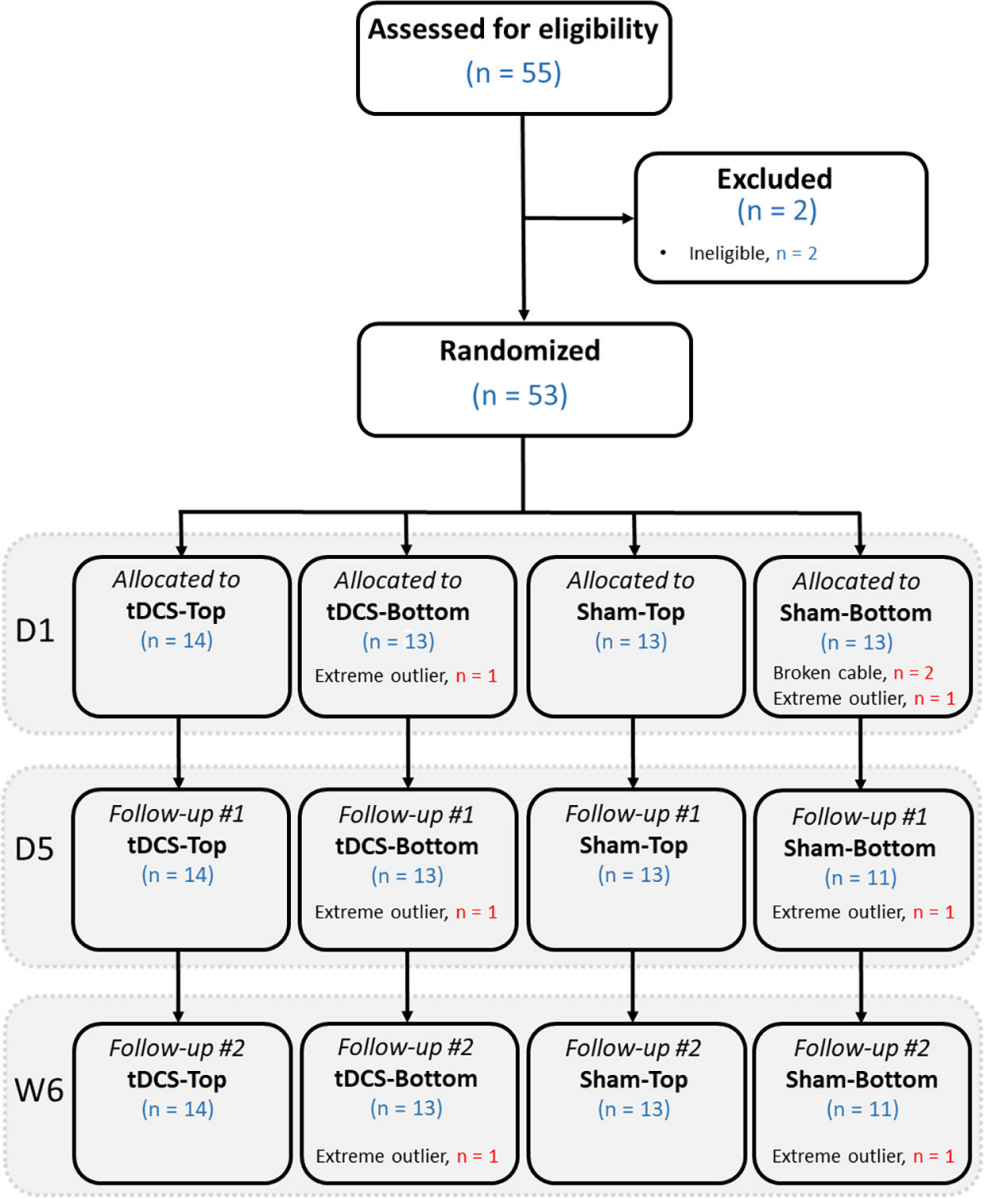
Flow diagram showing the number of participants at each stage, from eligibility assessment, randomization, allocation to the 4 groups, and follow-up. D1: day 1 (baseline); D5: day 5 (follow-up); W6: week 6 (follow-up).

The number of participants at each stage of the study is shown in Figure 3. In total, 55 participants were assessed for eligibility. Of these, 2 were found to be ineligible due to age or a history of head trauma. As a result, 53 participants proceeded to randomization. After group allocation, 2 in the Sham-Bottom group chose not to continue in the study after the stimulator cable broke on the first day. The final study sample consisted of 27 participants receiving real tDCS and 24 receiving sham tDCS. In the tDCS group, 14 participants were allocated to the tDCS-Top group and 13 to the tDCS-Bottom group. Thresholds for one participant in the tDCS-Bottom group were identified as extreme outliers across all time points and were excluded, leaving 12 participants in that group. In the sham group, 13 were in the Sham-Top group and 11 were in the Sham-Bottom group. One participant in the Sham-Bottom group was identified as an extreme outlier across all time points and were excluded, leaving 10 participants in that group. Table 1 presents the demographics and questionnaire scores of the study participants in the tDCS and Sham groups at baseline. No between-group differences were found in any of the parameters (Table 1). As expected, a high degree of right-hand dominance was found in each group. In addition, pain catastrophizing scores, anxiety scores, and depression scores were within normal range at the time of the experiment. Demographics and questionnaire scores from the subdivided groups are provided in Table 2. No statistical significant differences were observed among these groups.

**Table 1:** Demographics and questionnaire scores of the study participants in the two main groups (mean ± SD).

Characteristics	tDCS (*N* = 26)	Sham (*N* = 23)	*p*-value
tDCS configuration (Top/Bottom)	14/12	13/10	0.907^#^
Sex (Male/Female)	13/13	12/11	0.648^#^
Age	29.1 ± 6.9	28.3 ± 5.0	0.661*
Handedness (range: −1 to 1)	0.91 ± 0.21	0.91 ± 0.09	0.722*
PCS (0–52)	13.2 ± 10.3	10.8 ± 9.0	0.382*
STAI-S (range: 20 to 80)	40.6 ± 7.6	37.7 ± 6.9	0.180*
STAI-T (range: 20 to 80)	44.2 ± 8.5	40.3 ± 9.0	0.127*
CES-D (range: 0 to 60)	16.5 ± 8.6	13.7 ± 7.0	0.227*

CES-D, center for epidemiologic studies—depression scale; PCS, pain catastrophizing scale; STAI (S/T), Spielberger State–Trait anxiety inventory (State/Trait).

^#^ Chi-square test.

* Independent *t*-test.

**Table 2:** Demographics and questionnaire scores of the participants in the subdivided tDCS groups and in the sham group (mean ± SD).

Characteristics	tDCS-Top (*N* = 14)	tDCS-Bottom (*N* = 12)	Sham (*N* = 23)	*p*-value
Sex (Male/Female)	7/7	6/6	10/13	0.901^#^
Age	29.1 ± 7.3	29.1 ± 6.7	28.3 ± 5.0	0.909*
Handedness (range: −1 to 1)	0.89 ± 0.27	0.97 ± 0.04	0.91 ± 0.09	0.391*
PCS (0–52)	12.2 ± 9.0	14.4 ± 11.9	10.8 ± 9.0	0.581*
STAI-S (range: 20 to 80)	39.6 ± 8.6	41.7 ± 6.5	37.7 ± 6.9	0.321*
STAI-T (range: 20 to 80)	42.3 ± 8.7	46.5 ± 8.0	40.3 ± 9.0	0.150*
CES-D (range: 0 to 60)	15.2 ± 10.2	17.9 ± 6.4	13.7 ± 7.0	0.335*

CES-D, center for epidemiologic studies—depression scale; PCS, pain catastrophizing scale; STAI (S/T), Spielberger State–Trait anxiety inventory (State/Trait).

^#^ Chi-square test.

* One-way ANOVA.

### 3.2 Real tDCS vs. Sham stimulation

For PT threshold values, a data point was identified as an outlier at W6 in both the tDCS-Top and Sham-Top groups. Accordingly, the corresponding values at all time points were excluded from the within-group analysis for these participants. For MPT threshold values, outliers were identified at all three time points in one participant in the tDCS-Top group. This resulted in 25 participants in the combined tDCS group for both PT and MPT (13 in tDCS-Top and 12 in tDCS-Bottom), and 22 participants (12 in Sham-Top and 10 in Sham-Bottom) for PT, and 23 participants (13 in Sham-Top and 10 in Sham-Bottom) for MPT in the combined Sham group. In the within-group comparisons, where wire connector orientation was not considered, PT in the tDCS group showed a statistical difference after correcting for multiple comparisons (adjusted *p* = 0.008). *post-hoc* tests revealed significant increases between D1 and D5 (D5 > D1, adjusted *p* = 0.003), but not between D1 and W6 (adjusted *p* = 0.085) or between D5 and W6 (adjusted *p* = 0.774). MPT was also significant in the tDCS group (adjusted *p* = 0.004). Subsequent *post-hoc* tests revealed significant increases between D1 and D5 (D5 > D1, adjusted *p* = 0.003) and between D1 and W6 (W6 > D1, adjusted *p* = 0.011), but not between D5 and W6 (adjusted *p* = 1.000). Within-group comparisons for sham stimulation did not result in any significant findings (PT: adjusted *p* = 0.492; MPT: adjusted *p* = 0.852).

For changes in thresholds relative to baseline, 26 participants (14 tDCS-Top and 12 tDCS-Bottom) were included in the combined tDCS group for each threshold at each time point. In the Sham group, 23 participants (13 Sham-Top and 10 Sham-Bottom) were included for each threshold at each time point, except for PT at W6, where one data point was identified as an outlier and excluded, leaving 22 participants for that comparison. The tDCS and Sham groups, regardless of wire connector orientation, did not show any significant between-group differences in PT (tDCS, *n* = 26; Sham, *n* = 23; adjusted *p* = 1.000) or MPT (tDCS, *n* = 26; Sham, *n* = 23; adjusted *p* = 1.000) at baseline. Between-group differences were also not observed in relative threshold changes at D5 or W6 (PT D5: adjusted *p* = 0.680; PT W6: adjusted *p* = 1.000; MPT D5: adjusted *p* = 0.056; MPT W6: adjusted *p* = 0.584).

### 3.3 The effect of wire connector location

**Figure 4:**
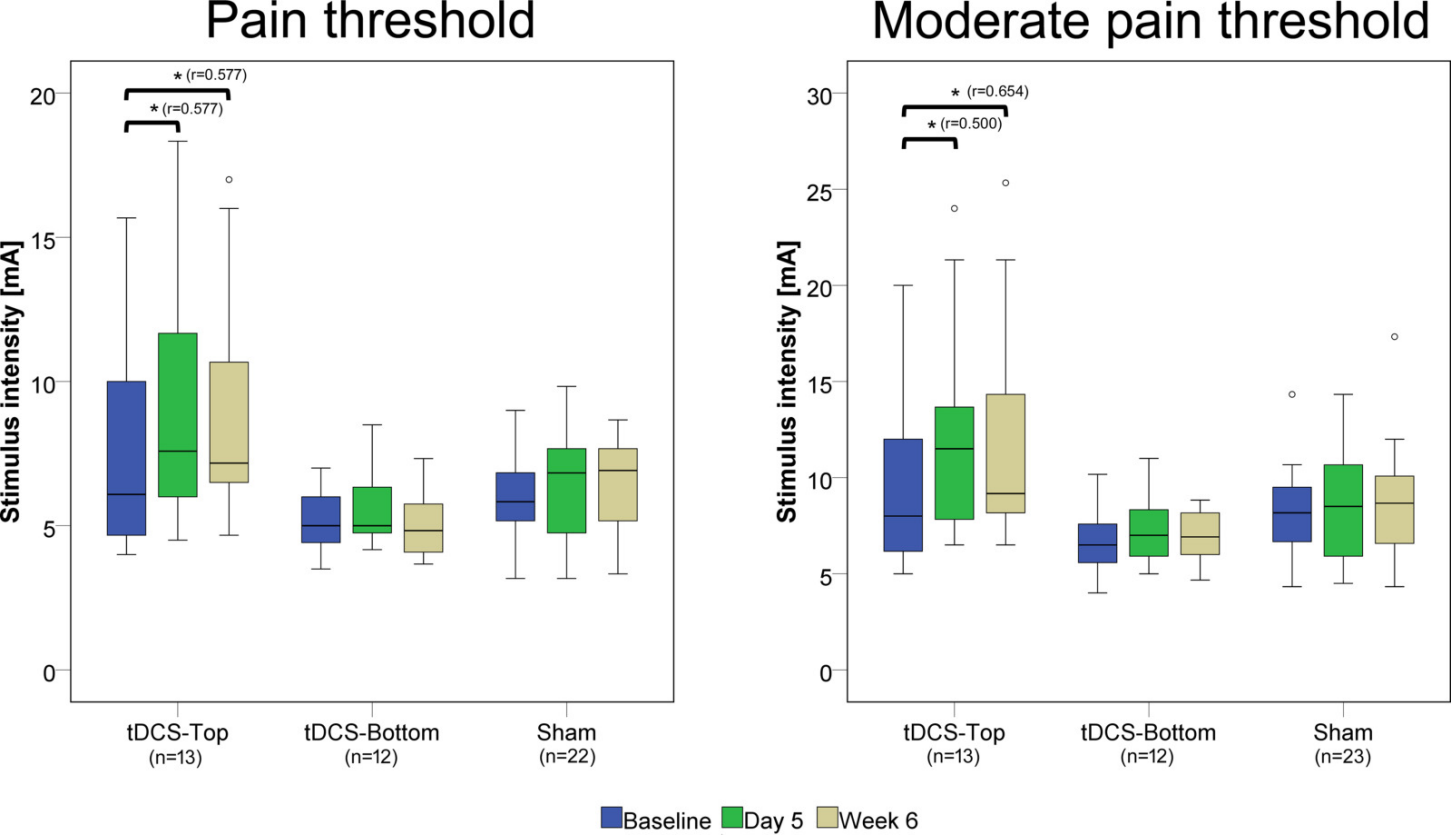
Box plots of pain detection thresholds and moderate pain thresholds. Thresholds were recorded before real or sham tDCS on the same day (D1), after 5 daily tDCS sessions (D5), and after 6 weeks (W6). Comparisons with statistically significant adjusted *p*-values are marked with an asterisk (*), and the effect size (r) is shown. The number of participants in each group is displayed below the group labels. Extreme outliers (defined as data points exceeding 3 times the interquartile range) were excluded from the plots. Mild outliers (values exceeding 1.5 times the interquartile range) are shown as individual data points.

The pain detection thresholds and moderate pain thresholds before and after tDCS are shown in Figure 4. Notably, thresholds in the tDCS-Top groups spanned a considerably wider range than in the other two groups, due to several participants with a high pain threshold. In the within-group comparisons between time points, both thresholds in the tDCS-Top group showed a statistical difference after correcting for multiple comparisons (adjusted *p* = 0.008). For PT, *post-hoc* tests revealed significant increases between D1 and D5 (D5 > D1, adjusted *p* = 0.010) and between D1 and W6 (W6 > D1, adjusted *p* = 0.010), but not between D5 and W6 (adjusted *p* = 1.000). Similarly, *post-hoc* tests for MPT revealed significant increases between D1 and D5 (D5 > D1, adjusted *p* = 0.032) and between D1 and W6 (W6 > D1, adjusted *p* = 0.003), but not between D5 and W6 (adjusted *p* = 1.000). Within-group comparisons for tDCS-Bottom did not result in any significant findings (PT: adjusted *p* = 0.480; MPT adjusted *p* = 0.404), suggesting that the observed effects in the combined tDCS group were primarily driven by changes within the tDCS-Top group.

**Figure 5:**
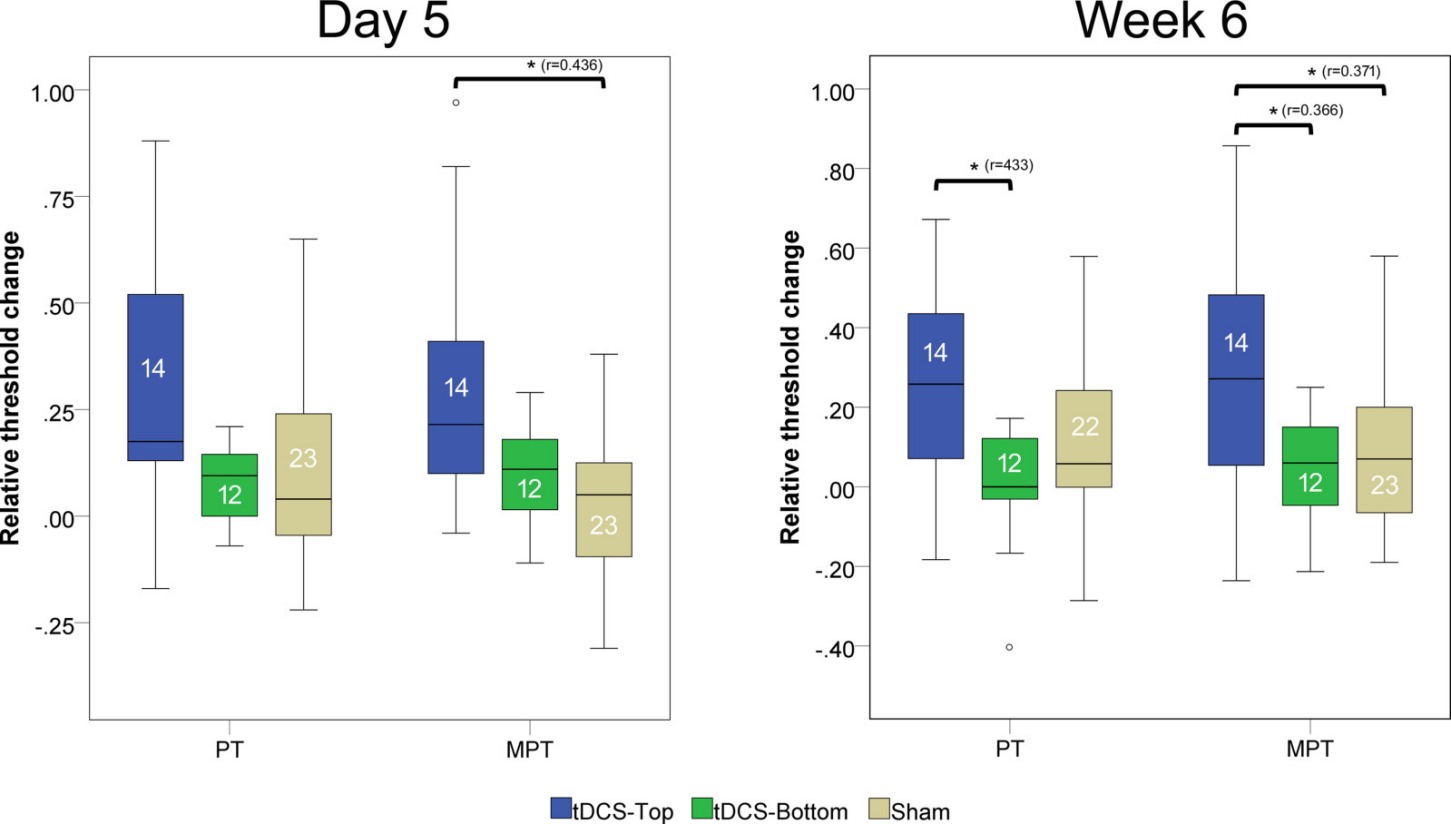
Box plot of changes in pain thresholds (PT) and moderate pain thresholds (MPT). Thresholds were recorded before tDCS (D1) and after 5 daily tDCS sessions(D5), and after 6 week follow up. The changes were defined as (D5-D1)/D1 and (W6-D1)/D1. Comparisons with statistically significant adjusted *p*-values are marked with an asterisk (*), and the effect size (r) is shown. The number of participants in each group is displayed within each box plot. Extreme outliers (defined as data points exceeding 3 times the interquartile range) were excluded from the plots. Mild outliers (values exceeding 1.5 times the interquartile range) are shown as individual data points.

Between-group comparisons of thresholds at baseline (D1) revealed no statistical differences (tDCS-Top: *n* = 14; tDCS-Bottom: *n* = 12; Sham: *n* = 23; PT: adjusted *p* = 0.242; MPT: adjusted *p* = 0.244). Figure 5 shows the relative changes in PT and MPT after real and sham tDCS, separately for D5 and W6. A significant group difference was found for MPT (adjusted *p* = 0.036), but not for PT (adjusted *p* = 0.256) at D5. For MPT, *post-hoc* tests revealed a significant difference between tDCS-Top and Sham (adjusted *p* = 0.007), but not between tDCS-Top and tDCS-Bottom (adjusted *p* = 0.210) or between tDCS-Bottom and Sham (adjusted *p* = 1.000). At W6, both thresholds resulted in significant differences (PT: adjusted *p* = 0.036; MPT: adjusted *p* = 0.048). *post-hoc* tests for PT revealed a significant difference between tDCS-Top and tDCS-Bottom (adjusted *p* = 0.008), but not between tDCS-Top and Sham (adjusted *p* = 0.102) or between tDCS-Bottom and Sham (adjusted *p* = 0.611). *post-hoc* tests for MPT revealed significant differences between tDCS-Top and Sham (adjusted *p* = 0.028) and between tDCS-Top and tDCS-Bottom (adjusted *p* = 0.031), but not between tDCS-Bottom and Sham (adjusted *p* = 1.000).

The 20% responder rates at D5 and W6 in the tDCS-Top group were 43% (*n* = 6) and 57% (*n* = 8) for PT, and 57% (*n* = 8) and 71% (*n* = 10) for MPT. In the tDCS-Bottom group, the corresponding rates were 15% (*n* = 2) and 8% (*n* = 1) for PT, and 17% (*n* = 2) and 8% (*n* = 1) for MPT. In the Sham group, responder rates were 30% (*n* = 7) and 28% (*n* = 6) for PT, and 22% (*n* = 5) and 30% (*n* = 7) for MPT.

### 3.4 GABA+ concentrations in Sm1

**Figure 6:**
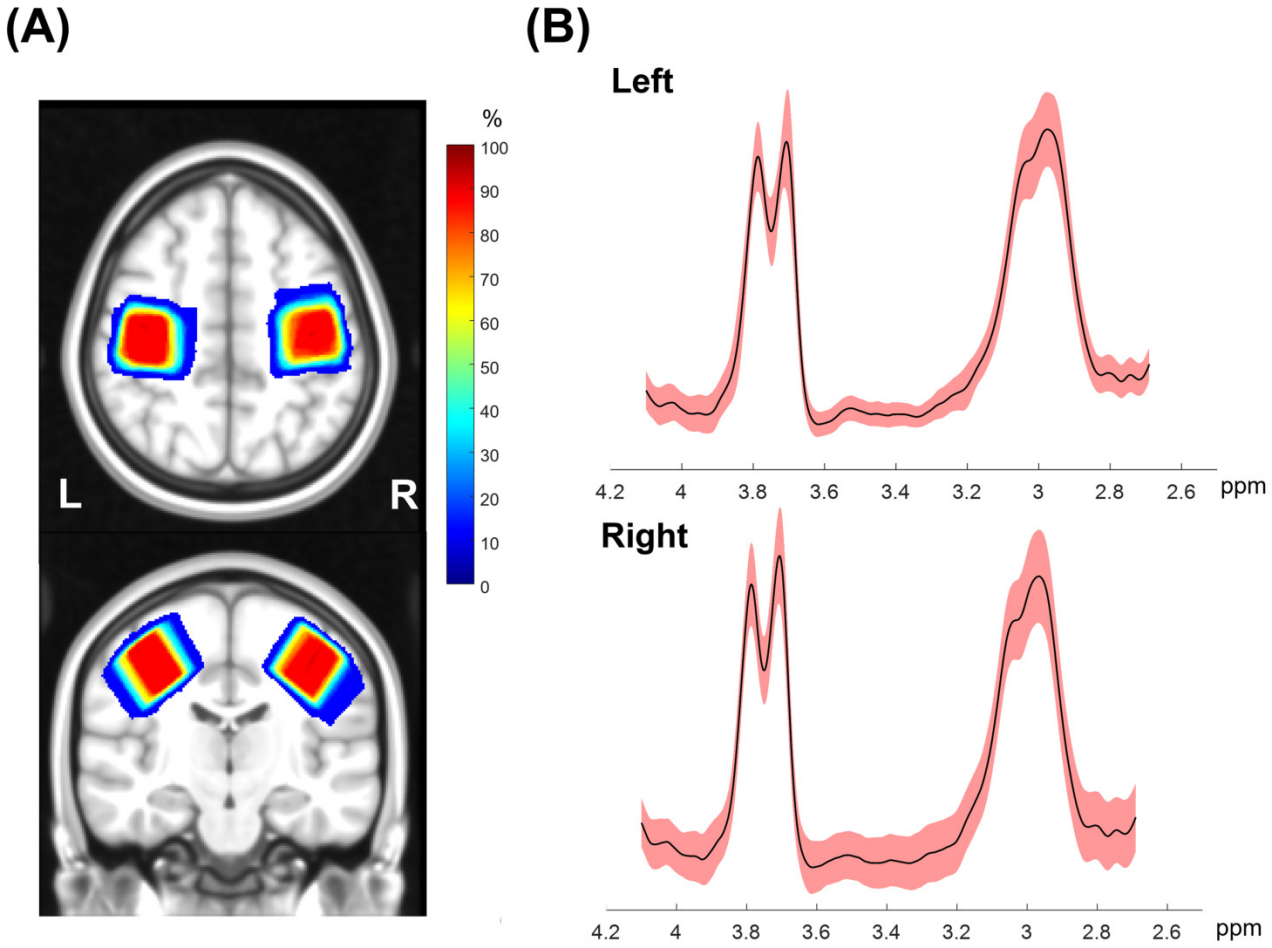
**(A)** Penetration maps showing the location of MRS voxels for left and right SMI across all subjects overlaid on the transverse and sagittal view of a template image in Montreal Neurological Institute standard space. Warm colors indicate higher overlap in location across subjects and dark red corresponds to an overlap of all subjects. **(B)** Mean (solid black) and standard deviation (shaded red) of edited GABA spectra from all subjects in a range between 2.7 to 4.1 ppm. The GABA+ peak at 3.0 ppm and Glx peak at 3.75 ppm can be identified in this spectral range.

Two participants in the tDCS-Top group underwent MRS scans only in the left SM1 due to technical problems. Table 3 summarizes the number of subjects included in the statistical analyses. Overall, spectra from the right SM1 showed lower spectral quality compared to those from the left SM1. Among the excluded data, one participant in the tDCS-Top group was entirely excluded due to poor spectral quality. Another seven spectra, all in the right hemisphere, were excluded due to linewidth criteria (*N* = 2), fitting error criteria (*N* = 3), or by visual inspection (*N* = 2), i.e., both displayed an asymmetric shape in the GABA signal and had small spurious peaks around the GABA peak. Figure 6A illustrates the location of the MRS VOIs across the remaining participants, overlaid on the mean anatomical image in standard space. The two VOIs consistently covered the hand area of the SM1s in all participants. Additionally, spectra from 2.7 ppm to 4.1 ppm for all participants are also displayed in Figure 6B. The fitting errors of GABA+ for the remaining participants were 4.54 ± 0.94% and 4.80 ± 1.12% for the left and right SM1, respectively. Additionally, the linewidth of the unsuppressed water signal was 9.18 ± 0.75 Hz and 9.24 ± 0.70 Hz for the left and right SM1, respectively. No between-group differences were found for any of the quality measures.

**Table 3:** Number of participants with valid spectra.

Configuration	Time point	Left GABA+	Right GABA+
tDCS-Top	Baseline	13	11
	Day 5	13	11
	Week 6	13	10
tDCS-Bottom	Baseline	12	12
	Day 5	12	12
	Week 6	12	11
Sham	Baseline	23	21
	Day 5	23	21
	Week 6	23	22

A total of 14, 12, and 23 participants were included in each sub-group for the study.

**Figure 7:**
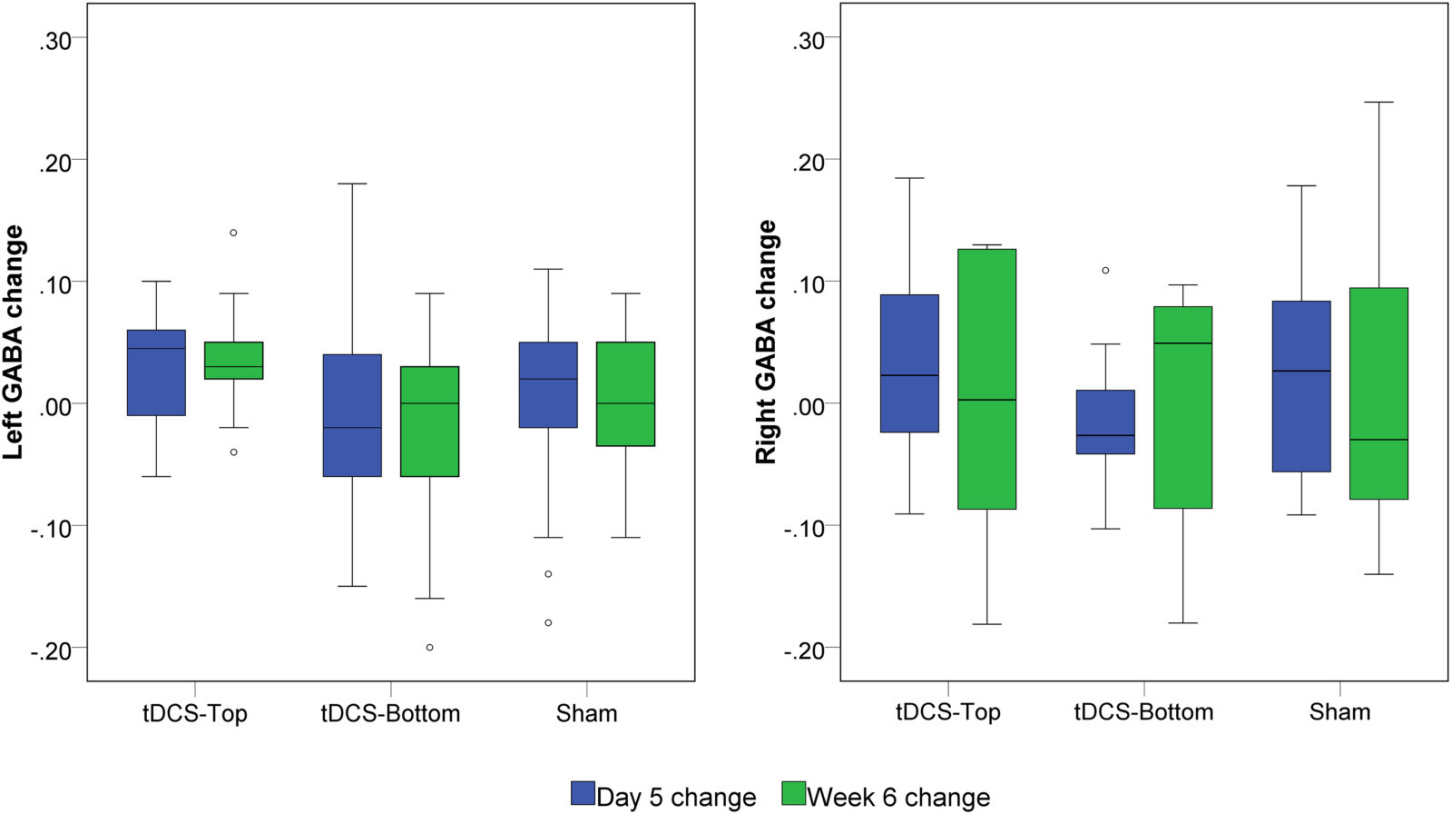
Box plot of left and right GABA+ changes. GABA+ was quantified before tDCS (D1), after 5 daily tDCS sessions (D5) and at six weeks (W6). The changes were defined as (D5-D1)/D1 and (W6-D1)/D1, respectively. No statistical significance was found among groups at both time points.

No within-group differences were found, whether the tDCS group was analyzed as a single group or divided into sub-groups, in both hemispheres (*p* > 0.199). Figure 7 depicts the change in bilateral GABA+ levels between D1 and D5, and between D1 and W6. For the left SM1, the changes from D1 to D5 and from D1 to W6 in the tDCS-Top group were 3.33 ± 4.56% and 2.35 ± 7.06%, respectively, which were higher than those in the tDCS-Bottom group (−0.94 ± 8.83% and −2.62 ± 8.55%) and the Sham group (0.19 ± 7.34% and 0.28 ± 5.77%). A similar tendency was not observed in the right SM1. Despite this, no statistical differences were found for any of the between-group comparisons of GABA+ levels in both hemispheres (adjusted *p* = 1.000). To examine whether baseline GABA+ levels in the stimulated hemisphere were predictive of the long-term outcome, Spearman's rank correlation analyses were conducted between left GABA+ concentrations and the change in pain thresholds at W6. As hypothesized, negative correlations were found in the tDCS-Top group for both PT [*r_s_* = −0.507, p(1-tailed) = 0.039] and MPT [*r_s_* = −0.492, p(1-tailed) = 0.044], but not for threshold changes in the other two groups [p(1-tailed) > 0.301]. However, none of the correlations passed an adjusted threshold (*p* = 0.0083).

### 3.5 Simulation of Top and Bottom montages

**Figure 8:**
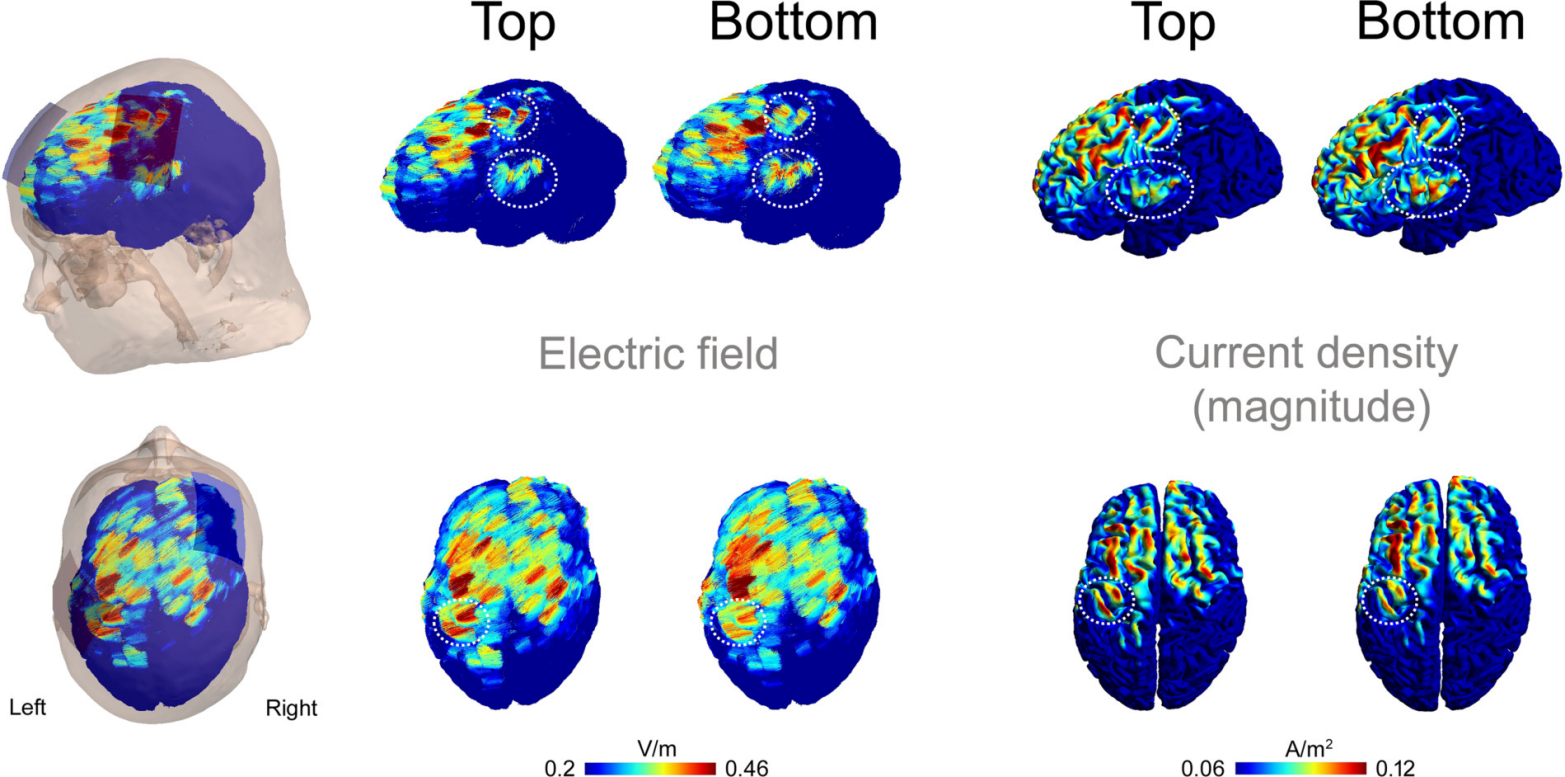
Electric fields and current densities resulting from simulations of the top and bottom montages. Results are shown in left (upper panels) and top (lower panels) views. Electric field vectors are overlaid, and the magnitude of the current density is projected onto the cortex. The encircled areas highlight regions of interest in the left SM1 where differences between the two montages were observed.

Figure 8 shows the electric fields and the magnitudes of the current densities resulting from simulations of the Top and Bottom montages. Notably, the Top configuration produced higher electric field strength and current density magnitudes compared to the Bottom configuration in the hand area of the precentral and postcentral gyri beneath the anode. This difference is most evident when the current density magnitudes are projected onto the cortex (Figure 8). In contrast, the Bottom configuration showed higher electric field and current density magnitude in the ventral portion of the SM1, as seen in the lateral view of Figure 8.

## 4 Discussion

The present study investigated whether the location of the wire connector on the anodal tDCS electrode influenced the modulation of pain sensitivity and the neuronal excitability in the stimulated SM1 cortex, as non-invasively measured with GABA+. Our findings revealed that anodal tDCS applied over the left SM1 using an electrode oriented superior-medially (tDCS-Top) significantly increased pain thresholds (PT and MPT) of the contralateral index finger, with effects lasting up to week 6. This effect was not observed with the ventral-lateral orientation of the anode (tDCS-Bottom) or with sham stimulation. Moreover, MPT at week 6 was significantly higher in the tDCS-Top group compared to both the tDCS-Bottom and sham groups. None of the tDCS configurations resulted in changes in GABA+ levels in either hemisphere, and no relationship was found between GABA+ and pain thresholds. Finally, differences in the simulated electric field and the magnitude of the current density were found in the hand area of SMI between the Top and Bottom configurations.

The stimulation regimen employed in the present study consisted of one tDCS session per day for 5 consecutive days. This protocol led to increased pain thresholds, i.e., reduced pain sensitivity, in the combined tDCS group immediately after the final tDCS session. Furthermore, the modulatory effect on suprathreshold pain could be sustained for at least 5 weeks following the end of tDCS. When the tDCS group was subdivided into tDCS-Top and tDCS-Bottom groups, these effects were observed only in the tDCS-Top group, where the wire connector of the anode was oriented in the superior-medial direction. This suggests that the effect observed in the combined group was primarily driven by the tDCS-Top group. Although previous studies have shown that cumulative tDCS effects can persist for weeks or months (8–10), this is the first demonstration that the orientation of the rectangular anode relative to the underlying cortex influences the modulatory effect on pain sensitivity. Contrary to our hypothesis, the modulatory effect was not observed in both anodal tDCS configurations when analyzed separately. Thus, the orientation of the rectangular anode during SM1 stimulation plays a significant role in the long-lasting modulation of perceived pain intensity. The between-group analysis further suggests that suprathreshold pain measures may be more sensitive indicators of tDCS-induced changes in pain sensitivity. Suprathreshold pain is likely more clinically relevant, as it better mirrors real-world scenarios, i.e., clinical pain is rarely experienced at pain detection level.

Several factors may explain the observed differences between the two anodal electrode orientations. First, the present study used rectangular electrodes (5 × 7 cm) with the wire connected at the far end. Previous modeling studies suggest that the highest electric field strength is located closer to the connector rather than at the geometric center of the electrode (13). In our simulation, we similarly observed that the local peak field strength and the peak magnitude of the current density differed between the tDCS-Top and tDCS-Bottom orientations. While peak values were higher dorsally for the tDCS-Top orientation, they were higher ventrally for the tDCS-Bottom orientation. This aligns with findings that even small deviations, on the order of 1 cm, can alter the distribution of current flow in the brain (3). Second, the behavioral and physiological effects of tDCS are influenced by the alignment between current flow and the orientation of neurons in the targeted cortex (3). Due to cortical folding, which can invert the direction of current flow locally (45), a slight shift in peak field location could result in distinct effects on pain sensitivity. A similar mechanism may explain why significant excitability changes in the motor cortex are observed only when the M1 electrode is angled at 45° to the midsagittal line along the central sulcus (12). Third, the topological organization of the SM1 must be considered. The C3 location in the 10–20 system is assumed to lie over the hand and finger representations, which cover a relatively large area extending superiorly. Just ventral to this region lies the face representation (46). As shown in our simulation, the peak electric field and current density in the tDCS-Top configuration remained within the hand representation area of SM1, whereas in the tDCS-Bottom configuration, it was located closer to the face representation, where pain thresholds were not assessed. Our findings may have implications for other tDCS montages using rectangular electrodes overlying regions where cortical heterogeneity exists along the long axis of the electrode. For example, positioning the connector anteriorly or posteriorly during DLPFC stimulation could lead to the activation of functionally distinct areas, such as Brodmann areas 9 and 6, respectively. In the same vein, this information could potentially be leveraged to optimize stimulation protocols, as in the present study where the connector was placed anteriorly. Further systematic investigations are needed to evaluate the sensitivity of behavioral outcomes to electrode orientation and positioning. Such studies could improve our understanding of the mechanisms underlying tDCS-induced pain modulation and help optimize its therapeutic application.

Although we investigated the modulation of acute pain evoked in healthy participants, our protocol and findings may have clinical relevance. It is well known that experimentally evoked pain in healthy individuals differs substantially from ongoing clinical pain, both in mechanisms and context. Nevertheless, several chronic pain conditions, such as fibromyalgia, neuropathic pain, and osteoarthritis, are characterized by hypersensitivity to evoked pain, i.e., hyperalgesia. It is conceivable that our tDCS protocol could reduce hyperalgesia in such patients. However, chronic pain conditions with prominent hyperalgesia often involve central sensitization, making it difficult to predict how our centrally acting protocol would affect these populations. An additional issue pertains to the interpretation of the primary outcome measures used in our study, which were the electrical current intensity (mA) required to produce constant levels of pain. Since subjective pain intensity was held constant across time points, changes in current intensity in response to tDCS reflect altered pain sensitivity rather than changes in perceived pain. At present, there is no standardized clinical interpretation for what constitutes a meaningful change in current intensity for electrical somatosensory stimulation. We found that 71% of participants receiving tDCS-Top stimulation required a ≥20% increase in current intensity to evoke moderate pain (MPT) at week 6. The corresponding proportions were 8% and 30% in the tDCS-Bottom and Sham groups, respectively. Furthermore, a 31% increase in the median stimulus intensity was required to evoke moderate pain in the tDCS-Top group compared to the pain detection threshold at baseline (PT: 6.1 mA; MPT: 8.0 mA). A 20% reduction in stimulus intensity may therefore correspond to a decrease of two or more points on the 0–10 numerical rating scale, which could be considered clinically meaningful. Further research involving chronic pain patients is necessary to confirm this assumption.

We did not observe any significant changes in GABA+ levels in either the left or right SM1 following five consecutive days of tDCS or at the six-week follow-up. Previous studies in healthy adults have reported acute decreases in GABA localized to the tDCS target area (7, 17, 18, 47). However, other studies have found no significant change in GABA levels after tDCS (48, 49). One plausible explanation for the lack of GABA changes in our study may relate to the stimulation paradigm used. Unlike most previous studies that employed single-session tDCS, we used a multiday protocol with delayed follow-up assessments. As GABA levels were not measured immediately after the first session, we cannot rule out the possibility that acute changes occurred but were not sustained. Furthermore, long-lasting modulatory effects on GABA may not mirror those seen after a single session, as different mechanisms may be involved. Specifically, long-term effects are thought to rely more on long-term potentiation (LTP)-like plasticity, which is glutamate-dependent (39, 50). Since the MRS method used in our study does not reliably quantify glutamate, it is conceivable that changes in excitatory neurotransmission contributed to the sustained effects observed. Overall, our findings suggest that the neurobiological mechanisms underlying longitudinal tDCS protocols may differ from those elicited by single-session tDCS.

The major limitation of the present study is the small sample size. It is likely that the study was underpowered to detect longitudinal changes in GABA+, given the substantial variability observed in GABA levels. This issue was further compounded by the exclusion of a larger proportion of data from the right SM1. Additional limitations related to the GABA+ measurements include the placement of the volume of interest (VOI) in SM1. Due to the curvature of the cortex, the cuboidal VOIs inevitably encompassed parts of both S1 and M1. Therefore, we refer to the voxel location as the primary sensorimotor cortex (SM1). Moreover, MRS cannot distinguish between extracellular and intracellular neurotransmitter concentrations, limiting interpretation of the underlying GABA+ mechanisms. Regarding pain threshold measurements, these were taken from the right index finger, corresponding to the dominant hand. However, it cannot be assumed *a priori* that similar effects would be observed with stimulation of the non-dominant hand. Interhemispheric inhibition may be stronger from the dominant to the non-dominant hemisphere than vice versa, as has been demonstrated in the motor system. Future studies are needed to explore this asymmetry. Finally, we acknowledge that this study was not preregistered, and the findings should therefore be interpreted with caution until replicated in preregistered studies.

In conclusion, anodal tDCS of SM1 contralateral to the stimulated hand may induce long-lasting changes in pain thresholds. This effect appears to depend on the location of the anodal wire connector when using rectangular electrodes. Specifically, greater pain modulation may be achieved when the connector is aligned in a superior-medial direction along the central sulcus.

## Data Availability

The original contributions presented in the study are included in the article/Supplementary Material. Further inquiries can be directed to the corresponding author.

## References

[1] Giannoni-Luza S Pacheco-Barrios K Cardenas-Rojas A Mejia-Pando PF Luna-Cuadros MA Barouh JL Noninvasive motor cortex stimulation effects on quantitative sensory testing in healthy and chronic pain subjects: a systematic review and meta-analysis. Pain. (2020) 161:1955–75. 10.1097/j.pain.0000000000001893 32453135 PMC7679288

[2] Vo L Ilich N Fujiyama H Drummond PD. Anodal transcranial direct current stimulation reduces secondary hyperalgesia induced by low frequency electrical stimulation in healthy volunteers. J Pain. (2022) 23:305–17. 10.1016/j.jpain.2021.08.004 34500109

[3] Woods AJ Antal A Bikson M Boggio PS Brunoni AR Celnik P A technical guide to tDCS, and related non-invasive brain stimulation tools. Clin Neurophysiol. (2016) 127:1031–48. 10.1016/j.clinph.2015.11.012 26652115 PMC4747791

[4] Boggio PS Zaghi S Lopes M Fregni F. Modulatory effects of anodal transcranial direct current stimulation on perception and pain thresholds in healthy volunteers. Eur J Neurol. (2008) 15:1124–30. 10.1111/j.1468-1331.2008.02270.x 18717717

[5] Kold S Graven-Nielsen T. Effect of anodal high-definition transcranial direct current stimulation on the pain sensitivity in a healthy population: a double-blind, sham-controlled study. Pain. (2021) 162:1659–68. 10.1097/j.pain.0000000000002187 33449508

[6] Nitsche MA Paulus W. Sustained excitability elevations induced by transcranial DC motor cortex stimulation in humans. Neurology. (2001) 57:1899–901. 10.1212/WNL.57.10.1899 11723286

[7] Stagg CJ Best JG Stephenson MC O’Shea J Wylezinska M Kincses ZT Polarity-sensitive modulation of cortical neurotransmitters by transcranial stimulation. J Neurosci. (2009) 29:5202–6. 10.1523/JNEUROSCI.4432-08.2009 19386916 PMC6665468

[8] Kang JH Choi SE Park DJ Xu H Lee JK Lee SS. Effects of add-on transcranial direct current stimulation on pain in Korean patients with fibromyalgia. Sci Rep. (2020) 10:12114. 10.1038/s41598-020-69131-7 32694653 PMC7374102

[9] Soler MD Kumru H Pelayo R Vidal J Tormos JM Fregni F Effectiveness of transcranial direct current stimulation and visual illusion on neuropathic pain in spinal cord injury. Brain. (2010) 133:2565–77. 10.1093/brain/awq184 20685806 PMC2929331

[10] Valle A Roizenblatt S Botte S Zaghi S Riberto M Tufik S Efficacy of anodal transcranial direct current stimulation (tDCS) for the treatment of fibromyalgia: results of a randomized, sham-controlled longitudinal clinical trial. J Pain Manag. (2009) 2:353–61. 21170277 PMC3002117

[11] Saturnino GB Siebner HR Thielscher A Madsen KH. Accessibility of cortical regions to focal TES: dependence on spatial position, safety, and practical constraints. Neuroimage. (2019) 203:116183. 10.1016/j.neuroimage.2019.116183 31525498

[12] Foerster A Yavari F Farnad L Jamil A Paulus W Nitsche MA Effects of electrode angle-orientation on the impact of transcranial direct current stimulation on motor cortex excitability. Brain Stimul. (2019) 12:263–6. 10.1016/j.brs.2018.10.014 30389333

[13] Saturnino GB Antunes A Thielscher A. On the importance of electrode parameters for shaping electric field patterns generated by tDCS. Neuroimage. (2015) 120:25–35. 10.1016/j.neuroimage.2015.06.067 26142274

[14] O'Connell NE Marston L Spencer S DeSouza LH Wand BM. Non-invasive brain stimulation techniques for chronic pain. Cochrane Database Syst Rev. (2018) 4:CD008208. 29652088 10.1002/14651858.CD008208.pub5 PMC6494527

[15] Antonenko D Thielscher A Saturnino GB Aydin S Ittermann B Grittner U Towards precise brain stimulation: is electric field simulation related to neuromodulation? Brain Stimul. (2019) 12:1159–68. 10.1016/j.brs.2019.03.072 30930209

[16] Bachtiar V Johnstone A Berrington A Lemke C Johansen-Berg H Emir U Modulating regional motor cortical excitability with noninvasive brain stimulation results in neurochemical changes in bilateral motor cortices. J Neurosci. (2018) 38:7327–36. 10.1523/JNEUROSCI.2853-17.2018 30030397 PMC6096041

[17] Bachtiar V Near J Johansen-Berg H Stagg CJ. Modulation of GABA and resting state functional connectivity by transcranial direct current stimulation. Elife. (2015) 4:e08789. 10.7554/eLife.08789 26381352 PMC4654253

[18] Kim S Stephenson MC Morris PG Jackson SR. tDCS-induced alterations in GABA concentration within primary motor cortex predict motor learning and motor memory: a 7T magnetic resonance spectroscopy study. Neuroimage. (2014) 99:237–43. 10.1016/j.neuroimage.2014.05.070 24904994 PMC4121086

[19] Niddam DM Wang SJ Tsai SY. Pain sensitivity and the primary sensorimotor cortices: a multimodal neuroimaging study. Pain. (2021) 162:846–55. 10.1097/j.pain.0000000000002074 32947544

[20] Oldfield RC. The assessment and analysis of handedness: the Edinburgh inventory. Neuropsychologia. (1971) 9:97–113. 10.1016/0028-3932(71)90067-4 5146491

[21] Sullivan MJL Bishop SR Pivik J. The pain catastrophizing scale: development and validation. Psychol Assess. (1995) 7:524–32. 10.1037/1040-3590.7.4.524

[22] Spielberger CD Gorsuch RL Lushene R Vagg PR Jacobs GA. Manual for the State-Trait Anxiety Inventory. Palo Alto, CA: Consulting Psychologists Press (1983).

[23] Radloff LS. The CES-D scale: a self-report depression scale for research in the general population. Appl Psychol Meas. (1977) 1:385–401. 10.1177/014662167700100306

[24] Yap JC Lau J Chen PP Gin T Wong T Chan I Validation of the Chinese pain catastrophizing scale (HK-PCS) in patients with chronic pain. Pain Med. (2008) 9:186–95. 10.1111/j.1526-4637.2007.00307.x 18298701

[25] Ma WF Liu YC Chen YF Lane HY Lai TJ Huang LC. Evaluation of psychometric properties of the Chinese mandarin version state-trait anxiety inventory Y form in Taiwanese outpatients with anxiety disorders. J Psychiatr Ment Health Nurs. (2013) 20:499–507. 10.1111/j.1365-2850.2012.01945.x 22762356

[26] Shek DTL. Reliability and factorial structure of the Chinese version of the state-trait anxiety inventory. J Psychopathol Behav Assess. (1988) 10:303–17. 10.1007/BF00960624

[27] Chin WY Choi EP Chan KT Wong CK. The psychometric properties of the center for epidemiologic studies depression scale in Chinese primary care patients: factor structure, construct validity, reliability, sensitivity and responsiveness. PLoS One. (2015) 10:e0135131. 10.1371/journal.pone.0135131 26252739 PMC4529142

[28] Jiang L Wang Y Zhang Y Li R Wu H Li C The reliability and validity of the center for epidemiologic studies depression scale (CES-D) for Chinese university students. Front Psychiatry. (2019) 10:315. 10.3389/fpsyt.2019.00315 31178764 PMC6537885

[29] Beissner F Brandau A Henke C Felden L Baumgartner U Treede RD Quick discrimination of A(delta) and C fiber mediated pain based on three verbal descriptors. PLoS One. (2010) 5:e12944. 10.1371/journal.pone.0012944 20886070 PMC2944851

[30] Boonstra AM Stewart RE Koke AJ Oosterwijk RF Swaan JL Schreurs KM Cut-off points for mild, moderate, and severe pain on the numeric rating scale for pain in patients with chronic musculoskeletal pain: variability and influence of sex and catastrophizing. Front Psychol. (2016) 7:1466. 10.3389/fpsyg.2016.01466 27746750 PMC5043012

[31] Mescher M Merkle H Kirsch J Garwood M Gruetter R. Simultaneous *in vivo* spectral editing and water suppression. NMR Biomed. (1998) 11:266–72. 10.1002/(SICI)1099-1492(199810)11:6<266::AID-NBM530>3.0.CO;2-J 9802468

[32] Davidson RJ. Anterior cerebral asymmetry and the nature of emotion. Brain Cogn. (1992) 20:125–51. 10.1016/0278-2626(92)90065-T 1389117

[33] Davidson RJ Irwin W. The functional neuroanatomy of emotion and affective style. Trends Cogn Sci. (1999) 3:11–21. 10.1016/S1364-6613(98)01265-0 10234222

[34] Chen J Zhou C Wu B Wang Y Li Q Wei Y Left versus right repetitive transcranial magnetic stimulation in treating major depression: a meta-analysis of randomised controlled trials. Psychiatry Res. (2013) 210:1260–4. 10.1016/j.psychres.2013.09.007 24113125

[35] Brunoni AR Moffa AH Sampaio-Junior B Borrione L Moreno ML Fernandes RA Trial of electrical direct-current therapy versus escitalopram for depression. N Engl J Med. (2017) 376:2523–33. 10.1056/NEJMoa1612999 28657871

[36] Caumo W Alves RL Vicuna P Alves C Ramalho L Sanches PRS Impact of bifrontal home-based transcranial direct current stimulation in pain catastrophizing and disability due to pain in fibromyalgia: a randomized, double-blind sham-controlled study. J Pain. (2022) 23:641–56. 10.1016/j.jpain.2021.11.002 34785366

[37] Mattoo B Tanwar S Bhatia R Tripathi M Bhatia R. Repetitive transcranial magnetic stimulation in chronic tension-type headache: a pilot study. Indian J Med Res. (2019) 150:73–80. 10.4103/ijmr.IJMR_97_18 31571632 PMC6798618

[38] Tanwar S Mattoo B Kumar U Bhatia R. Repetitive transcranial magnetic stimulation of the prefrontal cortex for fibromyalgia syndrome: a randomised controlled trial with 6-months follow up. Adv Rheumatol. (2020) 60:34. 10.1186/s42358-020-00135-7 32600394

[39] Monte-Silva K Kuo MF Hessenthaler S Fresnoza S Liebetanz D Paulus W Induction of late LTP-like plasticity in the human motor cortex by repeated non-invasive brain stimulation. Brain Stimul. (2013) 6:424–32. 10.1016/j.brs.2012.04.011 22695026

[40] Monte-Silva K Kuo MF Liebetanz D Paulus W Nitsche MA. Shaping the optimal repetition interval for cathodal transcranial direct current stimulation (tDCS). J Neurophysiol. (2010) 103:1735–40. 10.1152/jn.00924.2009 20107115

[41] Edden RA Puts NA Harris AD Barker PB Evans CJ. Gannet: a batch-processing tool for the quantitative analysis of gamma-aminobutyric acid-edited MR spectroscopy spectra. J Magn Reson Imaging. (2014) 40:1445–52. 10.1002/jmri.24478 25548816 PMC4280680

[42] Gasparovic C Song T Devier D Bockholt HJ Caprihan A Mullins PG Use of tissue water as a concentration reference for proton spectroscopic imaging. Magn Reson Med. (2006) 55:1219–26. 10.1002/mrm.20901 16688703

[43] Kreis R Boer V Choi IY Cudalbu C de Graaf RA Gasparovic C Terminology and concepts for the characterization of *in vivo* MR spectroscopy methods and MR spectra: background and experts’ consensus recommendations. NMR Biomed. (2020) 34:e4347. 10.1002/nbm.4347 32808407 PMC7887137

[44] Thielscher A Antunes A Saturnino GB. Field modeling for transcranial magnetic stimulation: a useful tool to understand the physiological effects of TMS? Annu Int Conf IEEE Eng Med Biol Soc. (2015) 2015:222–5. 10.1109/EMBC.2015.7318340 26736240

[45] Datta A Bansal V Diaz J Patel J Reato D Bikson M. Gyri-precise head model of transcranial direct current stimulation: improved spatial focality using a ring electrode versus conventional rectangular pad. Brain Stimul. (2009) 2:201–7e1. 10.1016/j.brs.2009.03.005 20648973 PMC2790295

[46] Penfield WR Rasmussen T. The Cerebral Cortex of Man: A Clinical Study of Localization of Function. New York: The Macmillan Company (1950).

[47] Antonenko D Schubert F Bohm F Ittermann B Aydin S Hayek D tDCS-induced modulation of GABA levels and resting-state functional connectivity in older adults. J Neurosci. (2017) 37:4065–73. 10.1523/JNEUROSCI.0079-17.2017 28314813 PMC6596583

[48] Nandi T Puonti O Clarke WT Nettekoven C Barron HC Kolasinski J tDCS induced GABA change is associated with the simulated electric field in M1, an effect mediated by grey matter volume in the MRS voxel. Brain Stimul. (2022) 15:1153–62. 10.1016/j.brs.2022.07.049 35988862 PMC7613675

[49] Nwaroh C Giuffre A Cole L Bell T Carlson HL MacMaster FP Effects of transcranial direct current stimulation on GABA and Glx in children: a pilot study. PLoS One. (2020) 15:e0222620. 10.1371/journal.pone.0222620 31910218 PMC6946135

[50] Agboada D Mosayebi-Samani M Kuo MF Nitsche MA. Induction of long-term potentiation-like plasticity in the primary motor cortex with repeated anodal transcranial direct current stimulation—better effects with intensified protocols? Brain Stimul. (2020) 13:987–97. 10.1016/j.brs.2020.04.009 32325264

