# Supplementary material for: Modulation of pain sensitivity by tDCS using different anodal connector locations: a single-blinded, randomized, sham-controlled study
Source: Front Pain Res (Lausanne). 2025 Jun 16;6:1533962. doi: 10.3389/fpain.2025.1533962 (PMC12206704; doi:10.3389/fpain.2025.1533962)
Supplement: Supplementary file 1 [file Datasheet1.docx]

**Supplementary Figure 1**

**
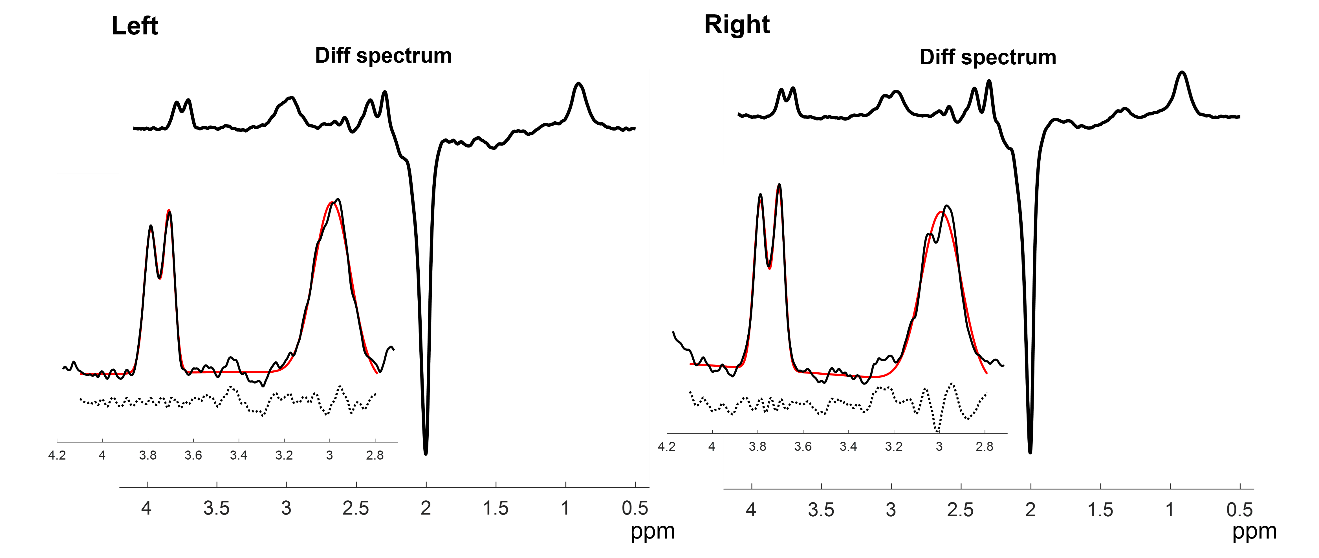
**

**Figure S1.** Representative GABA spectra of left and right SM1 from a subject. The diff spectrum was shown in the range of 4.2 ppm to 0.5 ppm. The fitted GABA and Glx peak (red line) at 3.0 ppm and 3.75 ppm were shown.
